# Supplementary material for: Disparities in telemedicine utilization among surgical patients during COVID-19
Source: PLoS One. 2021 Oct 8;16(10):e0258452. doi: 10.1371/journal.pone.0258452 (PMC8500431; doi:10.1371/journal.pone.0258452)
Supplement: S1 Table — (DOCX) [file pone.0258452.s001.docx]

**S1 Table. Sensitivity analysis excluding pediatric surgery patients of patient characteristics associated with a telemedicine visit during COVID-19, compared to pre-COVID in-person visits.**

| **Variable** | **Estimate** | **Standard Error** | **Odds Ratio** |
| --- | --- | --- | --- |
| Age, y | 0.002 | 0.003 | 1.00 |
| Female | 0.16 | 0.09 | 1.17 |
| Race/Ethnicity |  |  |  |
| Non-Hispanic White | 1 [Ref] |  |  |
| Non-Hispanic Black | 0.13 | 0.11 | 1.13 |
| Hispanic | -0.38 | 0.31 | 0.68 |
| Other/Unknown | 0.31 | 0.18 | 1.37 |
| English language preferred | 0.55 | 0.31 | 1.74 |
| Payer |  |  |  |
| Medicare | 1 [Ref] |  |  |
| Medicaid | 0.24 | 0.16 | 1.27 |
| Private | -0.005 | 0.11 | 1.00 |
| Other | 0.85 | 0.12 | 2.33 |
| DCI Group |  |  |  |
| Top-tier | 1 [Ref] |  |  |
| Mid-tier | -0.04 | 0.10 | 0.96 |
| Lower-tier | -0.28 | 0.10 | 0.76 |
| log(Distance, mi) | 0.01 | 0.05 | 1.01 |
| MyChart Activated | 0.33 | 0.09 | 1.40 |
| Specialty |  |  |  |
| Breast | 0.10 | 0.19 | 1.10 |
| Colorectal | -0.42 | 0.23 | 0.66 |
| Cardiothoracic | 0.21 | 0.19 | 1.24 |
| General | 1 [Ref] |  |  |
| MIS/Bariatric | -0.58 | 0.21 | 0.56 |
| Oncology | 0.32 | 0.20 | 1.37 |
| Transplant | 0.62 | 0.19 | 1.86 |
| Vascular | -0.95 | 0.23 | 0.39 |

DCI = distressed communities index, MIS = minimally invasive surgery
