# Supplementary material for: Disparities in telemedicine utilization among surgical patients during COVID-19
Source: PLoS One. 2021 Oct 8;16(10):e0258452. doi: 10.1371/journal.pone.0258452 (PMC8500431; doi:10.1371/journal.pone.0258452)
Supplement: S2 Table — (DOCX) [file pone.0258452.s002.docx]

**S2 Table. Sensitivity analysis excluding pediatric surgery patients of patient characteristics associated with an in-person visit during COVID-19, compared to in-person visits before COVID-19.**

| **Variable** | **Estimate** | **Standard Error** | **Odds Ratio** |
| --- | --- | --- | --- |
| Age, y | 0.008 | 0.004 | 1.01 |
| Female | 0.32 | 0.12 | 1.38 |
| Race/Ethnicity |  |  |  |
| Non-Hispanic White | 1 [Ref] |  |  |
| Non-Hispanic Black | 0.16 | 0.14 | 1.17 |
| Hispanic | -0.85 | 0.50 | 0.43 |
| Other/Unknown | -0.05 | 0.23 | 0.95 |
| English language preferred | 0.18 | 0.49 | 1.20 |
| Payer |  |  |  |
| Medicare | 1 [Ref] |  |  |
| Medicaid | 0.17 | 0.19 | 1.18 |
| Private | 0.06 | 0.14 | 1.06 |
| Other | 1.02 | 0.21 | 2.76 |
| DCI Group |  |  |  |
| Top-tier | 1 [Ref] |  |  |
| Mid-tier | -0.05 | 0.13 | 0.95 |
| Lower-tier | -0.22 | 0.12 | 0.80 |
| log(Distance, mi) | 0.06 | 0.07 | 1.06 |
| MyChart Activated | 0.15 | 0.11 | 1.16 |
| Specialty |  |  |  |
| Breast | 0.24 | 0.24 | 1.27 |
| Colorectal | -0.10 | 0.28 | 0.91 |
| Cardiothoracic | 0.49 | 0.25 | 1.63 |
| General | 1 [Ref] |  |  |
| MIS/Bariatric | -0.45 | 0.25 | 0.64 |
| Oncology | -0.04 | 0.24 | 0.96 |
| Transplant | 0.22 | 0.24 | 1.25 |
| Vascular | -0.90 | 0.29 | 0.41 |

DCI = distressed communities index, MIS = minimally invasive surgery
