# Supplementary material for: Disparities in telemedicine utilization among surgical patients during COVID-19
Source: PLoS One. 2021 Oct 8;16(10):e0258452. doi: 10.1371/journal.pone.0258452 (PMC8500431; doi:10.1371/journal.pone.0258452)
Supplement: S3 Table — (DOCX) [file pone.0258452.s003.docx]

**S3 Table. Sensitivity analysis excluding pediatric surgery patient characteristics associated with a telemedicine visit during COVID-19, compared to an in-person visit COVID-19.**

| **Variable** | **Estimate** | **Standard Error** | **Odds Ratio** |
| --- | --- | --- | --- |
| Age, y | -0.005 | 0.34 | 1.00 |
| Female | -0.11 | 0.07 | 0.90 |
| Race/Ethnicity |  |  |  |
| Non-Hispanic White | 1 [Ref] |  |  |
| Non-Hispanic Black | 0.05 | 0.09 | 1.05 |
| Hispanic | 0.24 | 0.21 | 1.27 |
| Other/Unknown | 0.40 | 0.13 | 1.49 |
| English language preferred | -0.04 | 0.22 | 0.96 |
| Payer |  |  |  |
| Medicare | 1 [Ref] |  |  |
| Medicaid | 0.02 | 0.11 | 1.02 |
| Private | -0.09 | 0.08 | 0.91 |
| Other | -0.28 | 0.14 | 0.75 |
| DCI Group |  |  |  |
| Top-tier | 1 [Ref] |  |  |
| Mid-tier | 0.02 | 0.08 | 1.02 |
| Lower-tier | -0.004 | 0.07 | 1.00 |
| log(Distance, mi) | -0.03 | 0.04 | 0.97 |
| MyChart Activated | 0.25 | 0.06 | 1.28 |
| Specialty |  |  |  |
| Breast | -0.06 | 0.15 | 0.94 |
| Colorectal | -0.22 | 0.15 | 0.80 |
| Cardiothoracic | 0.02 | 0.14 | 1.02 |
| General | 1 [Ref] |  |  |
| MIS/Bariatric | 0.13 | 0.14 | 1.14 |
| Oncology | 0.37 | 0.14 | 1.45 |
| Transplant | 0.53 | 0.15 | 1.70 |
| Vascular | -0.04 | 0.14 | 0.96 |

DCI = distressed communities index, MIS = minimally invasive surgery
