# Supplementary material for: Disparities in telemedicine utilization among surgical patients during COVID-19
Source: PLoS One. 2021 Oct 8;16(10):e0258452. doi: 10.1371/journal.pone.0258452 (PMC8500431; doi:10.1371/journal.pone.0258452)
Supplement: Attachment — Submitted filename: ResponsetoReviewers_2.docx [file pone.0258452.s005.docx]

September 23, 2021

Dear Editorial Staff of *PLOS ONE,*

We would like to thank the editor for their feedback and suggestions for our manuscript “Disparities in telemedicine utilization among surgical patients during COVID-19” (PONE-D-21-20375). We have made additional modifications to the manuscript and addressed each comment below.

As a result, we hope that the revised manuscript offers a stronger research contribution particularly in the wake of an ongoing pandemic.

Sincerely,

Courtney Lattimore, MD & Traci Hedrick, MD MS

Department of Surgery

P.O. Box 800709

Charlottesville, VA, 22908

Telephone: (434) 243-2670

Fax: (434) 924-2520

Journal Requirements:

*Please review your reference list to ensure that it is complete and correct. If you have cited papers that have been retracted, please include the rationale for doing so in the manuscript text, or remove these references and replace them with relevant current references. Any changes to the reference list should be mentioned in the rebuttal letter that accompanies your revised manuscript. If you need to cite a retracted article, indicate the article’s retracted status in the References list and also include a citation and full reference for the retraction notice.*

- The reference list was reviewed in its entirety and is both complete and correct with no retracted articles cited. A corrected citation was made in the Introduction (line 64, highlighted).

Additional Editor Comments (if provided):

*The authors properly addressed majority of the comments provided. However, a few additional edits need to be made to ensure accuracy of results reporting and conclusion drawing.*

*1. Abstract: page 2, Conclusions. Based on your findings, the current conclusions are not appropriately supported, because the significance of DCI (table 2) and female (table 4) came from two different models with different comparison groups. Please consider to use the revised Conclusions "During the first 3 months of the COVID-19 pandemic, Telemedicine was more likely used by females patients and those without government or commercial insurance compared to patients who used in-person visits. Interventions using telemedicine to improve health care access might consider such differences in utilization." Given this change, I suggest the authors to remove "socioeconomic" in their title.*

- Thank you for bringing this important point to our attention. The changes to the abstract have been made (lines 42-45, highlighted) and “Socioeconomic” has been removed from the title.

*2. Discussion, page 14, line 227-229: please consider revising this sentence to "Overall, telemedicine was more likely utilized by those without government or commercial insurance and women."*

- Thank you for this suggestion. The revision to this sentence has been made (lines 226-227, highlighted)

*3. Limitations, page 19. The authors only had access to less than 3 months of data after the COVID-19 outbreak, from March 17, 2020 to May 31, 2020. Please add a limitation of the early observation of the association based on the short period availability of data, which might be different from later into the pandemic.*

- We agree that the three months of data at the beginning of the pandemic are indeed a limitation. A statement to reflect this has been added (lines 334-337, highlighted).

*4. Conclusion, page 19, line 347. Please consider to change the sentence to "this study found that patient factors influence telemedicine utilization."*

- This change has been made. (line 347, highlighted)
